# Supplementary material for: Omega-3 Fatty Acids Upregulate SIRT1/3, Activate PGC-1α via Deacetylation, and Induce Nrf1 Production in 5/6 Nephrectomy Rat Model
Source: Mar Drugs. 2021 Mar 26;19(4):182. doi: 10.3390/md19040182 (PMC8066610; doi:10.3390/md19040182)
Supplement: Supplementary file 1 [file marinedrugs-19-00182-s001.pdf]

Omega-3 Fatty Acids Upregulate SIRT1/3, Activate PGC-1α via Deacetylation, and Induce Nrf1 Production in 5/6 Nephrectomy Rat Model

Sung Hyun Son [1,†], Su Mi Lee [2,†], Mi Hwa Lee [3], Young Ki Son [2], Seong Eun Kim [2] and Won Suk An [2,*]

1 Department of Internal Medicine, BHS Han Seo Hospital, Busan 48253 and Republic of Korea; sohnseung@hanmail.net
2 Department of Internal Medicine, Dong-A University, Busan 49201, Republic of Korea; sumilee@dau.ac.kr (S.M.L.); kidney@dau.ac.kr (Y.K.S.); sekim@dau.ac.kr (S.E.K.)
3 Department of Anatomy and Cell Biology, Dong-A University, Busan 49201, Republic of Korea; hero15p@nate.com
* Correspondence: anws@dau.ac.kr; Tel.: +82-51-240-2811
† These authors contributed equally to this work

Supplementary Information

A. Sham control group (serum creatinine 0.4 ± 0.0 mg/dL)

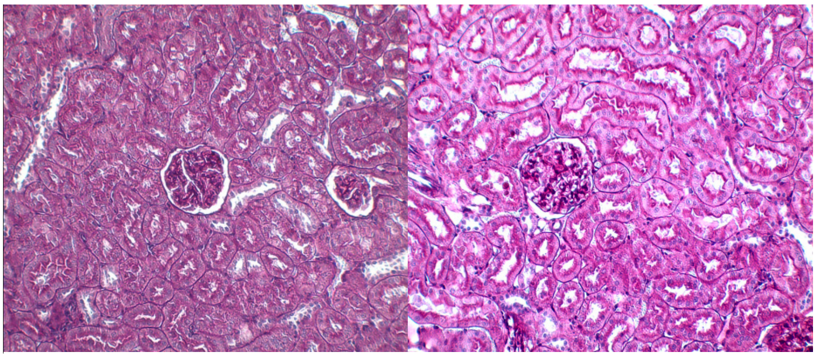

B. 5/6 Nephrectomy group (serum creatinine 1.3 ± 0.6 mg/dL)

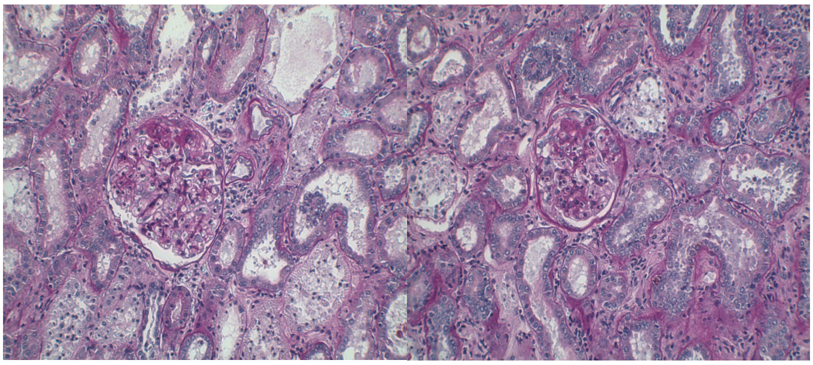

C. 5/6 nephrectomy treated with omega-3 fatty acid (serum creatinine 1.0 ± 0.3 mg/dL)

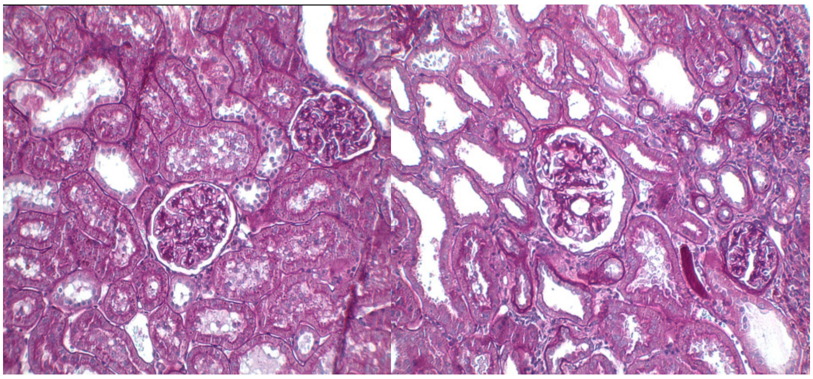

Figure S1 Morphological changes in the kidney among three groups representing renal function.

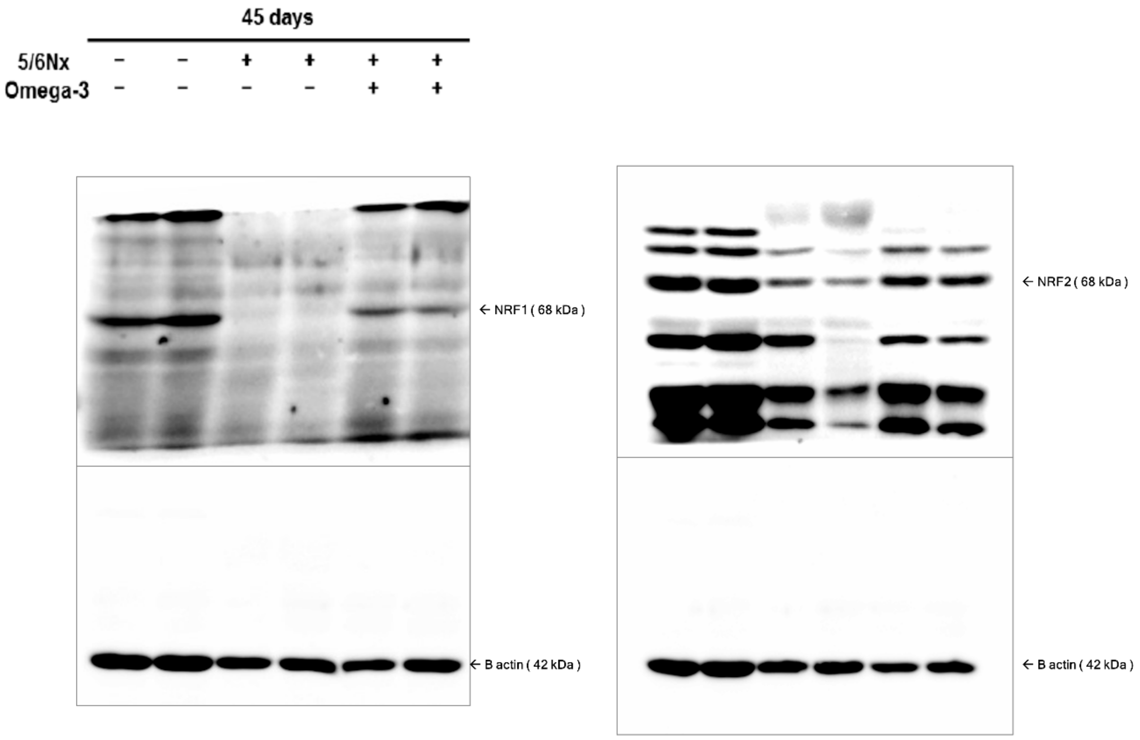

Figure S2. Expression of Nrf1 and Nrf2 on full length gels.

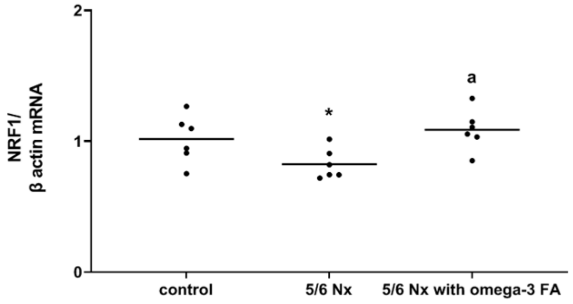

Figure S3 Expressions of Nrf1 by real time PCR among three groups. Decreased Nrf 1 mRNA of 5/6 nephrectomy group was improved by omega-3 FA supplementation. $*p$ value < 0.05 (mean values are significantly different from control). ^{a}p value < 0.05 (mean values are significantly different from the Nx group).

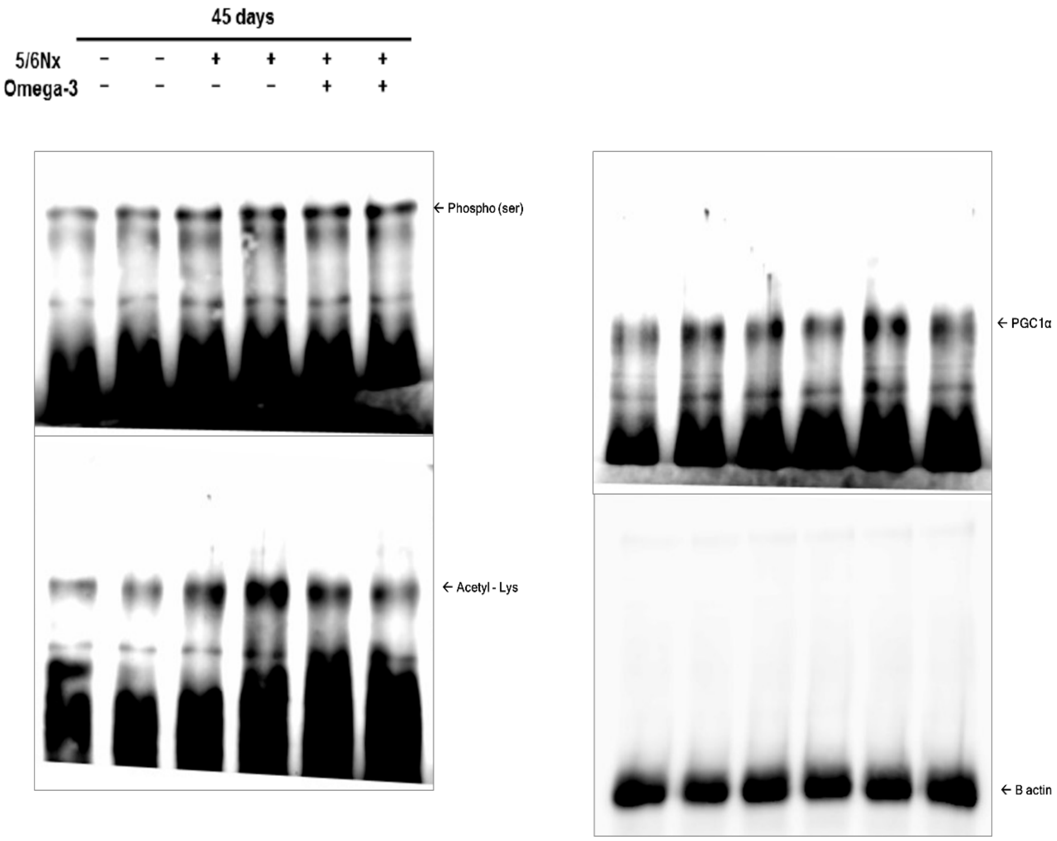

Figure S4 Expressions of phosphorylated PGC-1α, acetylated PGC-1α, and PGC-1α on full length gels.

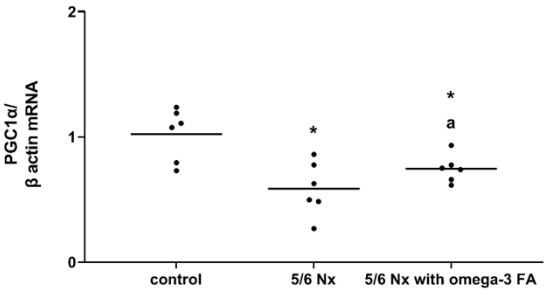

Figure S5 Expressions of PGC-1α by real time PCR among three groups. Decreased PGC-1α mRNA of 5/6 nephrectomy group was improved by omega-3 FA supplementation. *p value < 0.05 (mean values are significantly different from control). [a]p value < 0.05 (mean values are significantly different from the Nx group).

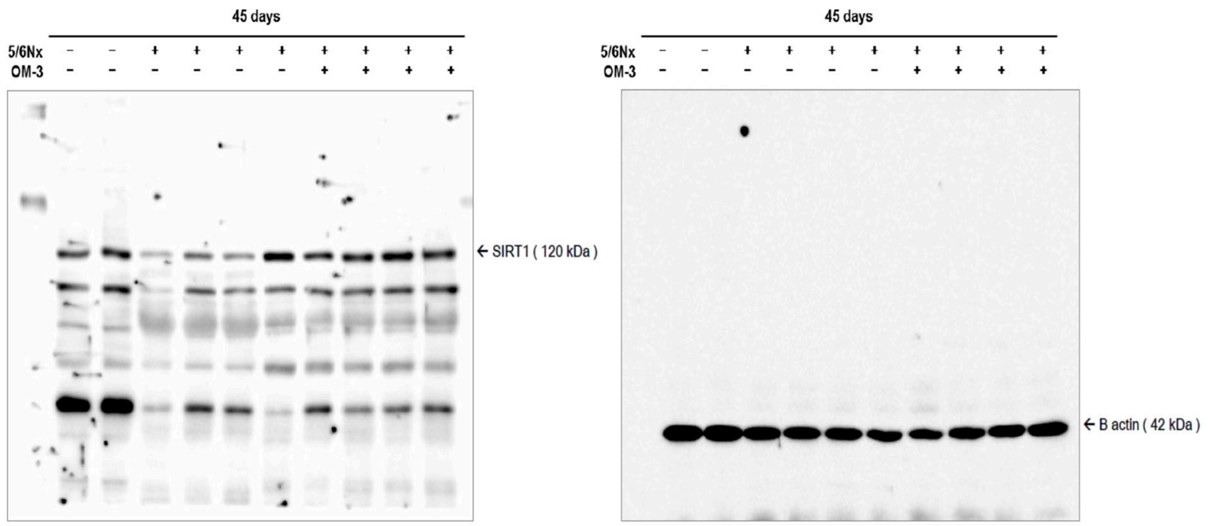

Figure S6 Expressions of SIRT1 on full length gels

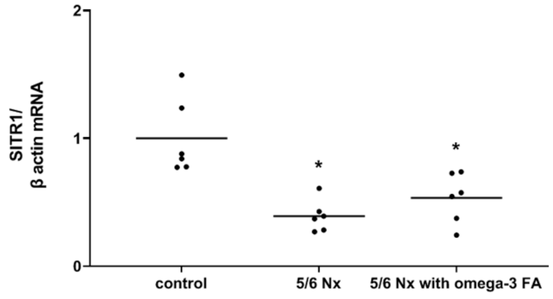

Figure S7 Expressions of SIRT1 by real time PCR among three groups. SIRT1 mRNA expression was similar with SIRT1 protein expression. **p* value < 0.05 (mean values are significantly different from control).

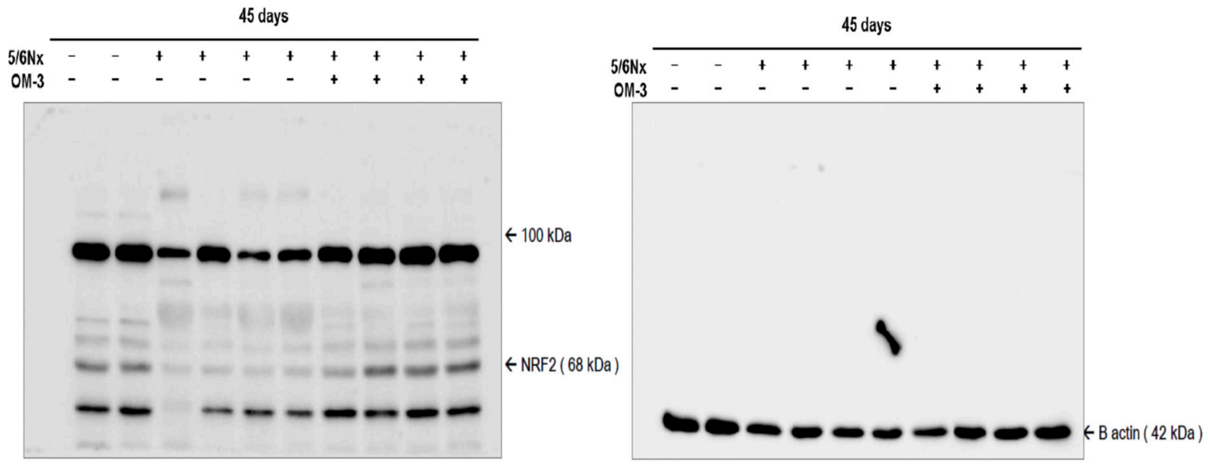

Figure S8 Expressions suspected as biologically relevant Nrf2 (96 kDa) on full length gels

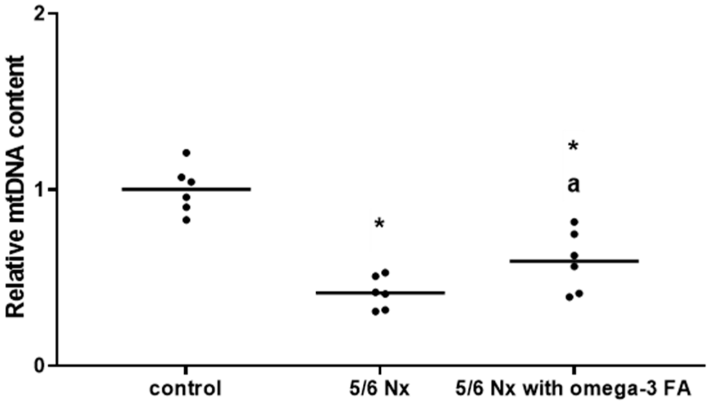

Figure S9 Relative content of mtDNA. *p value < 0.05 (mean values are significantly different from control). [a]P value < 0.05 (mean values are significantly different from the Nx group).
